# Reconciling intrinsic properties of activating TNF receptors by native ligands versus synthetic agonists

**DOI:** 10.3389/fimmu.2023.1236332

**Published:** 2023-09-19

**Authors:** George Fromm, Suresh de Silva, Taylor H. Schreiber

**Affiliations:** Shattuck Labs, Inc., Durham, NC, United States

**Keywords:** TNF superfamily, agonist, TNF receptor, TNF ligand, CD40, 41BB, OX40

## Abstract

The extracellular domain of tumor necrosis factor receptors (TNFR) generally require assembly into a homotrimeric quaternary structure as a prerequisite for initiation of signaling via the cytoplasmic domains. TNF receptor homotrimers are natively activated by similarly homo-trimerized TNF ligands, but can also be activated by synthetic agonists including engineered antibodies and Fc-ligand fusion proteins. A large body of literature from pre-clinical models supports the hypothesis that synthetic agonists targeting a diverse range of TNF receptors (including 4-1BB, CD40, OX40, GITR, DR5, TNFRSF25, HVEM, LTβR, CD27, and CD30) could amplify immune responses to provide clinical benefit in patients with infectious diseases or cancer. Unfortunately, however, the pre-clinical attributes of synthetic TNF receptor agonists have not translated well in human clinical studies, and have instead raised fundamental questions regarding the intrinsic biology of TNF receptors. Clinical observations of bell-shaped dose response curves have led some to hypothesize that TNF receptor overstimulation is possible and can lead to anergy and/or activation induced cell death of target cells. Safety issues including liver toxicity and cytokine release syndrome have also been observed in humans, raising questions as to whether those toxicities are driven by overstimulation of the targeted TNF receptor, a non-TNF receptor related attribute of the synthetic agonist, or both. Together, these clinical findings have limited the development of many TNF receptor agonists, and may have prevented generation of clinical data which reflects the full potential of TNF receptor agonism. A number of recent studies have provided structural insights into how different TNF receptor agonists bind and cluster TNF receptors, and these insights aid in deconvoluting the intrinsic biology of TNF receptors with the mechanistic underpinnings of synthetic TNF receptor agonist therapeutics.

## Introduction

The tumor necrosis factor (TNF) superfamily (TNFSF) of 19 ligands and 29 receptors serve as critical regulators of human immunity, and modulating the activity of individual receptors and ligands for therapeutic benefit in autoimmunity and cancer has been studied for over 40 years (1–3). Activating, or agonizing, TNF receptors to enhance immunity has proven to be a far more elusive goal than inhibiting TNF receptors. Enbrel (TNFR2-Fc) and Remicade (TNFα targeted monocolonal antibody [mAb]) were approved in 1998 to inhibit TNFα, and together with Humira (TNFα targeted mAb), quickly grew to become one of the most successful drug franchises in history (4, 5). In contrast, not a single TNF receptor agonist therapy (with the exception of recombinant TNFα) has progressed to a phase 3 clinical trial to date.

The focus of this review is to highlight the structural hypotheses underlying TNF receptor trimerization and subsequent activation of various cytoplasmic signaling cascades, both following activation via native TNF ligands and also with synthetic receptor agonists. A particular emphasis is placed upon areas where the pharmacodynamic activity of a TNF receptor agonist differed between pre-clinical mouse studies and human clinical trials. Several different TNF receptor agonists and TNF receptor targets, are included in this discussion, however the analysis is focused on how the available data inform on the magnitude and specificity of receptor engagement, rather than on the cellular and mechanistic differences between individual TNF receptors themselves. For example, this review focuses on whether the cytokine release syndrome (CRS) observed in human cancer patients treated with a 41BB or CD40 agonist antibody was likely a consequence of the underlying structural features of those antibody therapeutics rather than a deep dive into the specific differences in 41BB mediated costimulation of CD8 positive T cells versus CD40 mediated costimulation of antigen presenting cells (6–8).

Many patients, patient investors and drug developers have dedicated decades of effort to translating the powerful biology of TNF receptor agonists for the benefit of human disease. Most of these efforts have not lived up to the potential shown by the pre-clinical biology, yet many important lessons have been learned along the way. An improved understanding of the structural basis of TNF receptor activation has the potential to guide future development of improved therapeutic agonists.

## TNF receptor and ligand trimerization

All twenty nine TNF receptors, with the exception of DcR3, are single-pass type 1 membrane proteins, oriented with a cytoplasmic carboxy terminus and an extracellular amino terminus (9). DcR3 is a decoy receptor that evolved in higher-order primates as a secreted TNF receptor that functions as a soluble competitive inhibitor to LIGHT, TL1A and FasL (10). The extracellular domains of TNF receptors generally contain between one and four cysteine rich domains (CRDs), arranged in an elongated fashion within each monomer and which in turn are stabilized by a network of intrachain disulfide bridges. A majority of the interactions between TNF receptors and their ligands tend to involve the membrane-distal CRDs, and ligand binding occurs both through hydrophobic and polar interactions (9, 11).

Nineteen distinct TNF ligands exist in humans, and all are single-pass type 2 membrane proteins, oriented with a cytoplasmic amino terminus and an extracellular carboxy terminus (9). A conserved c-terminal TNF homology domain (THD) characterizes each TNF ligand, and mediates interaction with conserved cysteine rich domains (CRDs) in corresponding TNF receptors. The THD domain is arranged as a series of two stacked β-pleated sheets. The inner β-sheet contains the contact sites which mediate predominantly hydrophobic interactions between TNF ligand monomers, and contribute to assembly of stable TNF ligand homotrimers. The outer surface of the β-sheet structure mediates binding to the CRDs of the cognate TNF receptors (9). An underexplored aspect of TNF ligand trimerization relates to the conditions under which TNF ligand trimers assemble and degrade, and whether other cellular or matrix components are involved in the process. For example, TRAIL homotrimers were reported to assemble around a central Zn^2+^ ion, however it is unknown whether other TNF ligand trimers are similarly dependent upon cation coordination (9, 12).

In the absence of ligand, a full-length TNFR exists at the cell membrane as a mixture of monomers and dimers, whereas soluble TNFR exist primarily as monomers. Quantitative high resolution microscopy studies of cells with physiological expression of TNFR1 demonstrated that 66% of TNFR1 molecules are present as monomers and 34% are present as dimers (13–15). Following stimulation with ligand, the balance shifts to 13% TNFR1 monomers, 64% trimers, and 23% higher-order oligomers (15). Dimerization of TNFR can occur primarily as a result of non-covalent, low-affinity, interactions between pre-ligand assembly domains (PLAD), which are typically in a low micromolar affinity range (16–18). Ligand-induced trimerization of TNFR is likely influenced by a variety of non-covalent interactions, including the PLAD domains, but the quantum of signaling transmitted by the cytoplasmic domains increases when ligand-induced avidity interactions lead to trimerization, hexamerization, and higher-order network formation such as the 9-mers observed for TNFR1 (19–21).

The hypothesis that the efficiency of TNF receptor signaling is related to the degree of hexamer or higher-order network formation in the cell membrane is supported by several mechanistic studies. A minority of TNFR (including BaffR, DR3, GITR, LTβR and TNFR1) achieve activation with soluble ligand trimers, and are referred to as Category 1 TNFR. Clinical data are available for GITR agonist antibodies, but not for any of the others. For the so-called Category 2 TNF receptors (including 41BB, CD40, OX40, and others), soluble ligand trimers fail to activate downstream receptor signaling, unless those ligand trimers are cross-linked either via an Fc domain fused to the amino terminus of the TNF ligand extracellular domain, or if the soluble ligand trimers are cross-linked by an anti-TNF ligand antibody (9, 22). That the minimal signaling unit of TNF receptors is a trimer is a logical extension of the observation that the TNF receptor associated factor (TRAF) cytoplasmic adaptor complexes also require assembly into trimers to initiate signaling. Thus, a trimerized clover-like TNF ligand complex engages and facilitates trimerization of a TNF receptor complex in order to recruit and facilitate trimerization of cytoplasmic TRAF signaling adaptor complexes. Whether or not trimerization of TNF receptors is driven primarily by ligand-induced proximity interactions or via an associated conformational change in the structure of individual TNF receptor monomers is unclear. The structural basis of a ‘resting’ versus ‘active’ state of individual TNF receptors could be influenced by a transition between low-affinity interactions between neighboring TNF receptor monomers via the PLAD domains to higher-affinity interactions in the presence of trimerized ligand. Another possibility includes the association between TNF receptors and other accessory molecules, such as galectin-9, which could influence both affinity interactions between adjacent TNF receptor monomers, or potentially higher-order avidity interactions in a higher-order network (11, 23). A corresponding higher-order structural model of TRAF family oligomerization has been reported, wherein the ultimate signal-transduction potential of TNF receptor activation would be proportional both to the number of functional membrane trimers which are engaged by ligand, and also the degree to which those trimers assemble into an approximated higher-order network (9, 24).

Experimental evidence therefore consistently demonstrates that TNF receptor signaling is facilitated by TNF ligand mediated oligomerization of TNF receptors into homotrimeric complexes, which may then form higher-order 6-mer, 9-mer and potentially higher-order networks in cell membranes (15, 21). Transitioning this understanding to synthetic agonists with *in vivo* activity has proven to be an elusive goal, however. Over the years the number of synthetic agonist compounds has expanded, and now includes: monoclonal IgG antibodies, bispecific antibodies, tetravalent antibodies, hexavalent antibodies, Fc-fusion proteins, anticalin fusion proteins, bispecific Fc-fusion proteins, and IgM antibodies (**Table 1**) (3, 22, 25–27, 29, 32–34, 36, 37). All of these synthetic agonists have reported activity in pre-clinical models, particularly when the model facilitates an ‘array’ of individual agonist molecules, but the translatability of that pre-clinical data to *in vivo* activity in human patients has been dismal. One contributing factor to this lack of translatability may be related to the ability of different types of synthetic agonists to facilitate higher-order clustering of TNF receptors, which are further discussed in the following sections.

**Table 1:** Molecular configurations of synthetic TNFR agonists.

Type of TNFR Agonist		Molecular Configuration	Valency to TNFR	Compounds that have entered clinical trials	References
Antibody formats	Monoclonal IgG	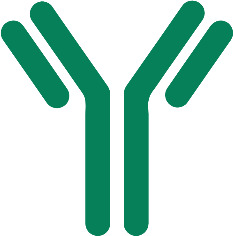	2	BMS-986178 PF-04518600 ADC-1013 BMS-986156	(3, 23, 25–28) Described in **Figures 1**–**3**
	Bispecific	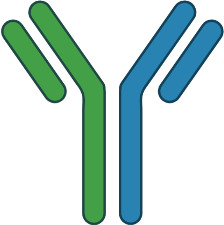	1	GEN1042 GEN1046 FS222 PRS-343	(29) Described in **Figure 4**
	Tetravalent	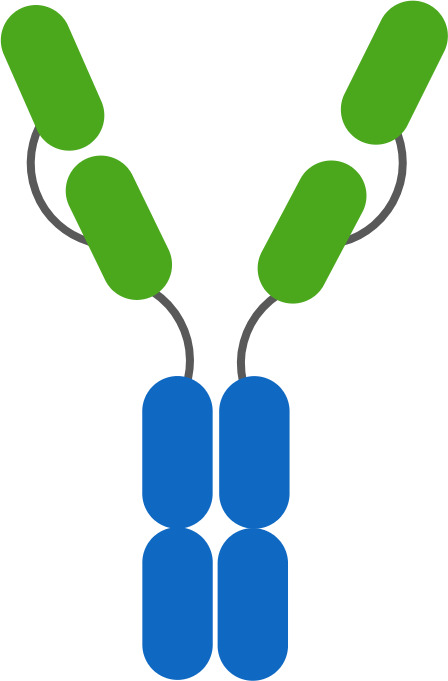	4	TAS266 INBRX-109	(29)
	Hexavalent	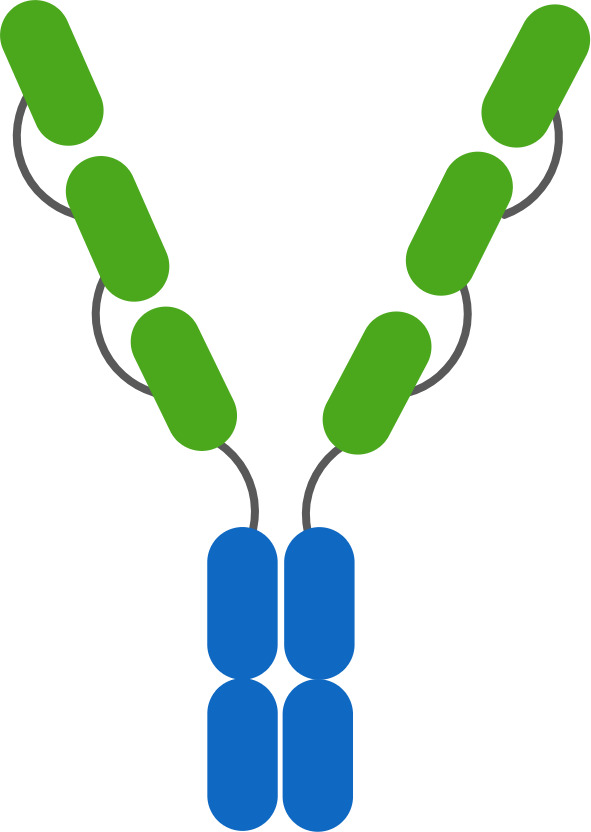	6	ABBV-621 and INBRX-106	(3) Described in **Figure 5**
	IgM	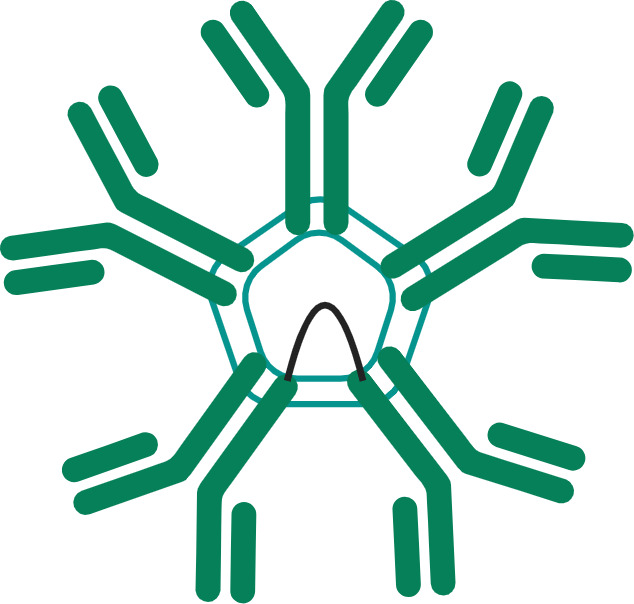	5	IGM-8444	(3)
Fc-fusion protein formats	Contains TNF ligand trimer	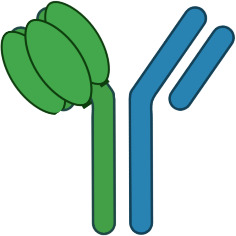	3	RO7227166 RO7122290	[30, 31) Described in **Figure 6**
	Bispecific (hexameric TNF ligand)	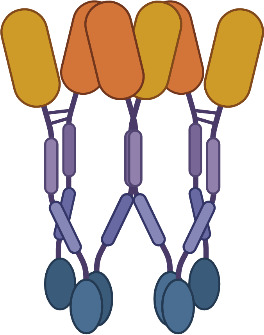	6	SL-279252 SL-172154 MEDI6383 MEDI1873	(32–35) Described in **Figure 7**

## Clinical data from trials testing bivalent TNFR agonist bivalent antibodies

A majority of synthetic TNF receptor agonists which entered clinical trials were IgG based monoclonal antibodies being tested in oncology indications, and the most common targets in those trials included: CD40, OX40, 41BB, GITR and DR5. Dadas et al. published a recent review of these approaches, which provides a helpful background on some of the TNFR targets and a thorough description of the role played by the Fc domain of different agonist antibodies (35). The focus of the following section is thus to synthesize how the available human clinical data reflect the underlying mechanisms of mAb based agonists.

All TNF receptor agonist monospecific antibodies tested in clinical trials incorporate Fc domains with retained Fc gamma receptor (FcγR) binding activity, as this was shown to be a pre-requisite for agonist activity in most cases (28). The most common Fc domain for clinical stage agonist mAbs is IgG1, followed by IgG2, with only a few developers selecting the IgG4 isotype (38, 39). Whereas the FcγR binding activity of most targeted antibodies is required for effector function (antibody dependent cellular phagocytosis:ADCP and/or antibody dependent cellular cytotoxicity:ADCC), agonist antibodies depend on FcγR binding purely as a mechanism to immobilize antibodies such that the TNF receptor binding ends of multiple mAbs are displayed as an array on the FcγR expressing cell. FcγRIIB provides particularly efficient cross-linking of TNF receptor agonist mAbs, but other FcγR can also participate (21, 40, 41). Mouse antibodies, particularly IgG1, bind to FcγRIIB with high affinity, whereas human antibodies have universally low affinity for FcγRIIB (40, 42). One of the general observations which can be made from reviewing the last thirty years of literature on TNF receptor agonist antibodies is that the *in vivo* activity of TNFR agonist antibodies has been much more potent in mouse syngeneic models than in human clinical trials, and this relative difference in binding affinity to FcγRIIB may be a significant contributor to that learning, particularly in light of pre-clinical data showing that only a two-fold reduction in the binding affinity to FcγRIIB was sufficient to eliminate the agonist activity of several TNFR agonist mAbs (43).

**Figure 1:**
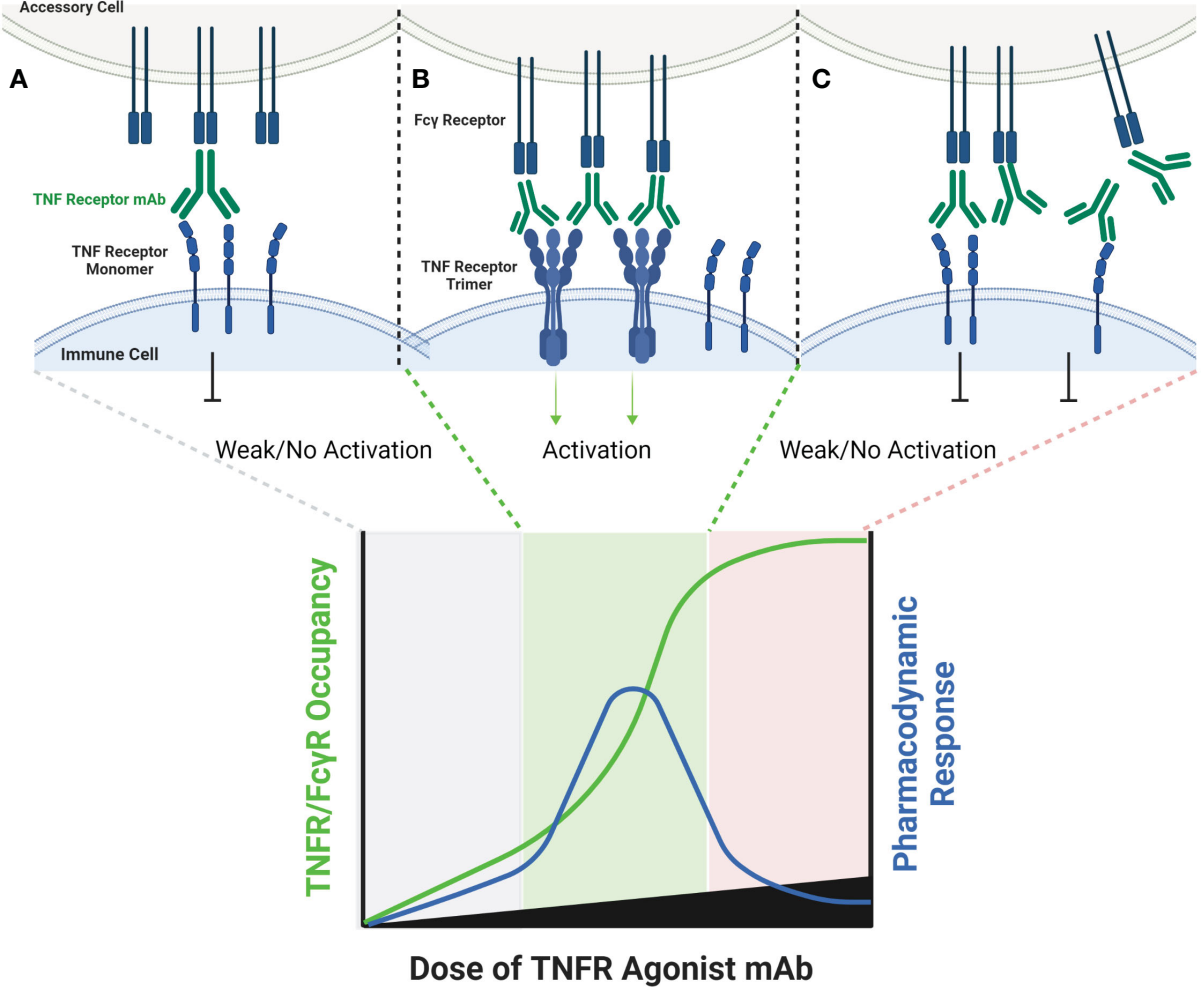
Schematic of FcγR Dependent TNFR Agonist mAb Dose Response. TNFR most commonly exist as monomers and dimers in cell membranes, and can be bound by one or both scFv domains of bivalent mAbs **(A)**. TNFR agonist mAbs commonly require FcγR binding for TNFR activation to occur, which is dependent upon the Fc domain of the TNFR agonist mAb binding to FcγR on an ‘accessory cell’ so that multiple TNFR agonist mAbs can be displayed as an array when binding TNFR **(B)**. An array of multiple TNFR agonist mAbs may include 4, 6, 8, etc. scFv domains in close proximity, capable of approximating the corresponding number of TNFR on a target cell if those TNFR are unoccupied. Administration of saturating concentrations of a TNFR mAb can result in a reduction in the number of free TNFR or FcγR **(C)**. If a TNFR is bound by a TNFR agonist mAb, then that TNFR is not available to bind TNFR agonist mAbs which have been ‘arrayed’ via FcγR binding, thus reducing activation of TNFR and the corresponding pharmacodynamic response.

The FcγR-dependent mechanism of TNFR agonist antibodies requires sub-saturating receptor occupancy on both the TNFR target and Fcγ receptors. As illustrated in **Figure 1**, FcγR dependent TNFR agonist antibodies must engage both target TNFR and FcγR simultaneously to promote antibody-mediated TNFR clustering in a trans orientation. This mechanism thus inherently depends upon having both free Fcγ receptor and free TNFR. The probability that FcγR bound mAbs encounter free TNFR target follows a Gaussian distribution, where a ‘maximal’ effect is predicted to occur when approximately 50% of the TNFR targets remain unoccupied by mAbs, assuming that both the abundance of and binding affinity to both TNFR target and Fcγ receptor are similar for a particular antibody (44). Gaussian distribution curves can also be described to have a ‘bell-shaped’ appearance, which is in fact the way in which the pharmacodynamic effects of many TNFR agonist antibodies tested in clinical trials have been reported. Here, high doses of TNFR agonist mAbs can independently saturate the intended TNFR and also the FcγR required to facilitate TNFR clustering, thereby eliminating agonist activity.

Amongst the clearest examples of bell-shaped dose response effects in humans treated with TNF receptor agonist antibodies include data from cancer patients treated with BMS-986178 (anti-OX40 mAb), PF-04518600 (anti-OX40 mAb) or mitazalimab (anti-CD40 mAb). Each of these antibodies are dependent on FcγR mediated cross-linking for agonist activity. The main pharmacodynamic marker reported from patients treated with the two OX40 agonist mAbs was proliferation (as indicated by Ki67 expression) of specific T cell subsets (44, 45). In both studies, a greater fold change in the proportion of Ki67+ T cells was observed in the low and mid-dose groups (~2 mg/kg or lower) than in the higher dose groups. The fold change observed in humans was also generally 3-fold or less, whereas the fold change in pre-clinical studies was approximately 5-fold or greater (44). Neither study reported corresponding changes in the actual numbers of the T cell subsets that stained positive for Ki67 expression. A phase 1 study tested mitazalimab across a dose range of 0.075-1.2 mg/kg in patients with advanced solid tumors (46), and reported a broader range of pharmacodynamic findings (47). Specifically, deep and rapid declines in the number of B cells in the peripheral blood were reported following the first dose, which was attributed to migration of B cells which are known to express CD40, and presumed to be bound by mitazalimab. Another finding included increased serum concentrations of multiple chemokines, including MCP1, IP10, MIP1α and MIP1β (46). The dose response for each of these chemokines showed a peak increase at the 0.075 or 0.2 mg/kg dose level, and lower magnitude increases in each chemokine at doses of 0.9 and 1.2 mg/kg. Wang et al. then proposed a model for the dosing of BMS-986178, wherein the optimal pharmacodynamic activity was achieved when the dose of the OX40 agonist mAb achieved approximately 50% receptor occupancy on OX40 expressing T cells (44).

A combination of inter-patient and intra-patient variability in TNF receptor abundance creates a significant challenge to selecting a single dose to advance into larger clinical trials if the mechanism of TNF receptor agonist mAbs requires sub-maximal receptor occupancy for the desired biological effect, as described above. Consider ‘Patient A’, who has a PBMC count of 1.4x10^6^ cells per mL, of which 80% are lymphocytes, 85% of the lymphocytes are T cells, 60% of the T cells are CD4+ T cells and 20% of those CD4+ T cells express OX40. Also consider ‘Patient B’, who has a PBMC count of 0.8x10^6^ cells per mL, of which 70% are lymphocytes, 70% of the lymphocytes are T cells, 50% of the T cells are CD4+ T cells and 5% of the CD4+ T cells express OX40. In both patients, these example lymphocyte/T cell percentages fall within the ‘normal’ range for healthy adults (the magnitude of variability can have a wider range in oncology patients who have received prior chemotherapy), yet Patient A will have approximately 10^5^ CD4+OX40+ T cells per mL of peripheral blood, and Patient B will have approximately 10^4^ CD4+OX40+ T cells per mL of peripheral blood. If the dose of an OX40 agonist antibody is modeled on the basis of achieving 50% receptor occupancy, the appropriate doses for Patient A and Patient B would differ by 10-fold. In a phase 1 clinical trial of SL-279252, we observed even wider variation, with the number of CD4+ T cells ranging from 2.2x10^3^ to 1.1x10^6^, and the percentage of CD4+OX40+ cells ranging from 5.1-48.6% (48, 49). Further, the example above assumes that each CD4+OX40+ cell expresses the same number of OX40 receptor molecules, which is unlikely to be the case either at baseline or through a course of therapy. In fact, both preclinical and clinical studies have reported that individual antigen-specific CD4+OX40+ T cell clones can expand more than 5-fold following treatment with an OX40 agonist, and the per-cell expression of OX40 can also increase following stimulation (13, 50, 51). Thus, a dose of an OX40 agonist antibody that achieves 50% receptor occupancy at the first dose is unlikely to be an appropriate dose several weeks later if activation of OX40+ cells has actually occurred, because both the density of OX40 on the cell surface, and the absolute number of CD4+OX40+ T cells would be expected to increase. The above example is not intended to indicate that inter- and intra- patient variability in degree to which a selected dose of a TNFR agonist agent is likely to cross an ‘all or none’ threshold of activation, but rather to highlight the risk that a selected dose for an agent with an expected bell-shaped dose response curve may lead to variable degrees of TNFR activation which may or may not remain in a therapeutic range.OX40 agonism is one such example, but the same mechanistic dependencies can likely be generalized to other TNF receptors including 4-1BB, CD27, CD40, GITR and others.

Additional clinical evidence is available to support this assertion from GITR agonist antibody studies, the only Category I TNFR target from which clinical data are available. GITR, as a Category 1 TNFR, is activated by soluble ligand trimers, and therefore it may be expected that bivalent antibodies would more readily activate this TNFR given the lack of a mechanistic requirement for higher-order TNFR oligomer assembly. A phase 1 clinical trial testing BMS-986156 (IgG1 Fc domain) in late stage cancer patients did not report any clear dose-dependent pharmacodynamic activity following infusion, either alone or in combination with nivolumab. In a phase 1 clinical trial with an FcγR non-binding GITR agonist, TRX518, some evidence reductions in regulatory T cells were reported both in the peripheral blood and within the tumor. The dose-dependence of this effect was not clear from the study given the limited sample size, nor was the potential mechanism of action given that GITR lacks a death domain.

Another variable to consider in selecting an optimal dose for a TNF receptor agonist antibody - based on sub-maximal receptor occupancy - involves the potential competition for Fcγ receptor binding. Two pre-clinical studies concluded that the sequencing of an OX40 agonist antibody and a PD-1 inhibitory antibody determined whether or not the combination was efficacious, despite not controlling for the fact that both antibodies were the same isotype, and thus competed with one another for Fcγ receptor binding (16, 17). The clinical impact of this specific combination is limited, given that pembrolizumab and nivolumab both have inactive IgG4 Fc domains, however there are many potential antibody combinations where it could be relevant. This antibody/antibody FcγR interference issue becomes an even greater challenge to contend with in clinical trials, due to the fact that most human or humanized antibodies have long half-lives and can be detected at therapeutically relevant levels in serum for greater than 6 months following discontinuation of therapy.

**Figure 2:**
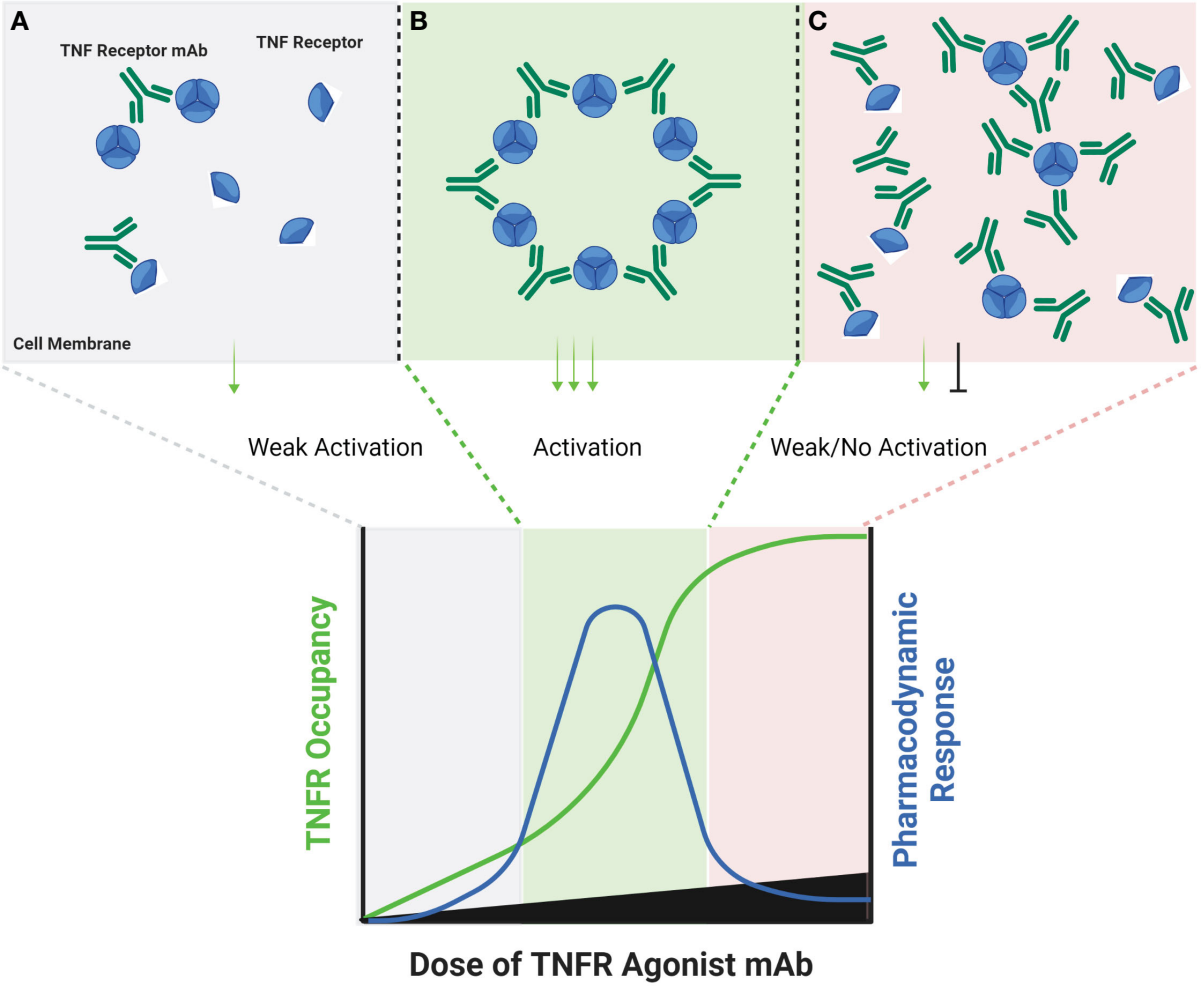
Schematic of FcγR Independent Clustering of TNFR Networks in Cell Membranes. Certain TNFR exist in cell membranes as inactive dimers, which can then assemble into trimers upon interaction with a corresponding TNF ligand. Certain TNFR agonist mAbs, may be capable of stimulating a similar response via binding particular epitopes on adjacent TNFR in cell membranes. At low antibody concentrations, TNFR agonist mAbs may bind epitopes on adjacent TNFR, and sometimes cause activation by approximating TNFR dimers or trimers into higher-order networks **(A)**. A hypothetical maximum response is predicted to occur in this model when the molar ratio or TNFR agonist mAb is equal to the number of available binding sites on each trimer of a target TNFR **(B)**. When this ratio is reached, every TNFR trimer is theoretically cross-linked to another TNFR trimer by the TNFR agonist mAb, thus creating a high-order network of TNFR. When the concentration of TNFR agonist mAb exceeds the number of available epitopes on TNFR dimers and trimers, then a TNFR bound by one arm of an antibody may not lead to cross-linking with a nearby TNFR, if that nearby TNFR is also bound by one or both arms of another TNFR agonist mAb **(C)**, thus reducing receptor activation and the corresponding pharmacodynamic responses.

**Figure 3:**
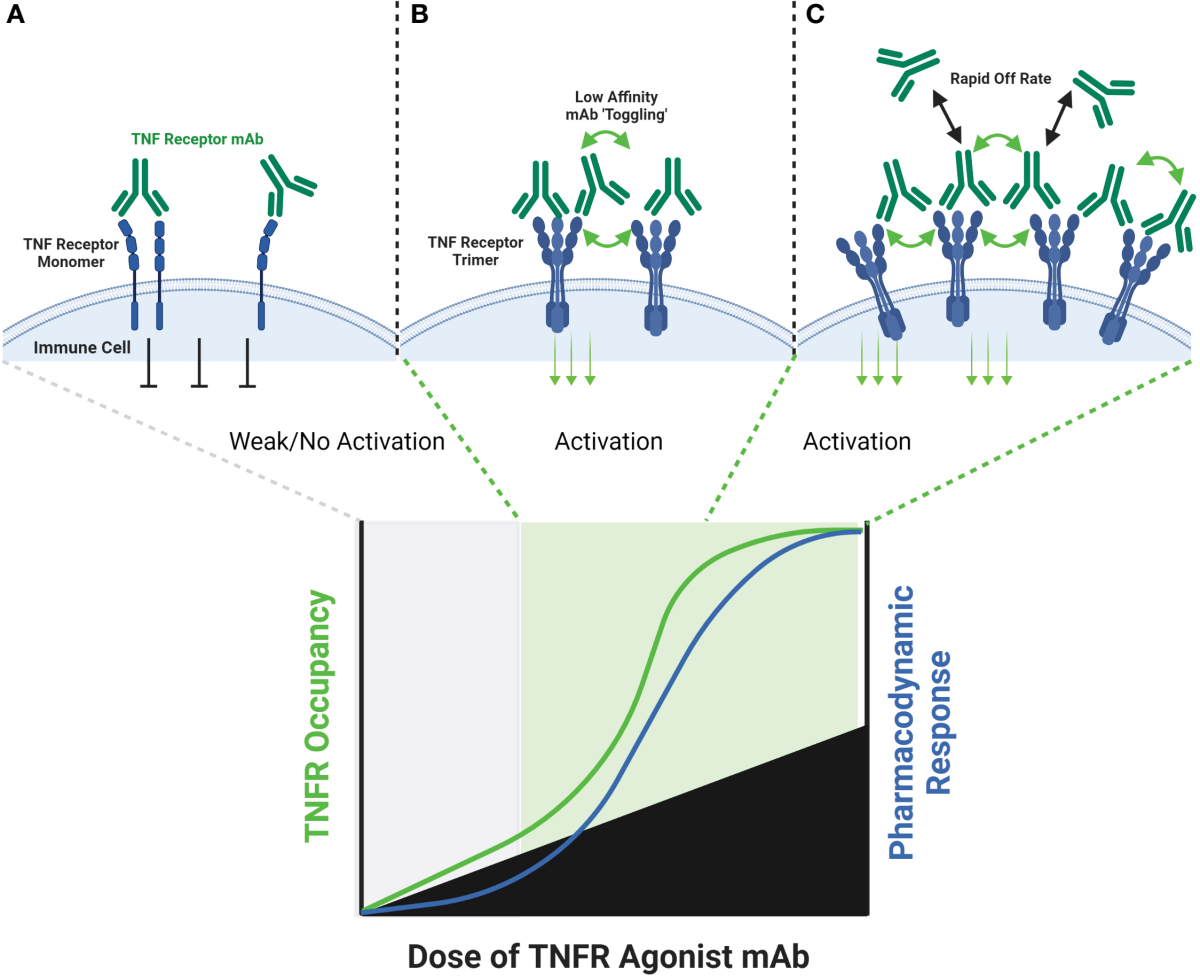
Schematic of FcγR Independent Clustering of TNFR by Low-Affinity TNFR Agonist mAbs. Both high affinity and low affinity TNFR agonist mAbs are capable of binding a TNFR monomer or dimer in a similar manner, however the low affinity antibody will have a faster ‘off-rate’ than the high affinity antibody **(A)**. The faster off-rate of low affinity antibodies leads to an equilibrium where antibodies are rapidly binding and releasing a TNFR target in a cell membrane. In some cases, one arm of the antibody could remain bound to a TNFR while the other arm releases and then re-binds another adjacent TNFR, leading to clustering and TNFR activation **(B)**. If the off-rate of a low affinity antibody is fast enough, then a persistent state of ‘receptor occupancy’ may not occur. This property could theoretically enable low-affinity antibodies to toggle on-and-off a TNFR target fast enough to cause cross-linking and activation even when the molar ratio of the TNFR agonist mAb is in excess to the number of TNFR binding sites **(C)**.

Some TNF receptor agonist antibodies have been described as ‘super agonists’, which is a descriptive term indicating that the functional activity of the antibody is independent on FcγR binding. Recent work has provided important insights into the potential mechanisms of action for super agonist antibodies, and suggest they relate to a combination of the specific epitope bound by the antibody, and the binding affinity of the antibody. Vanamee and Faustman proposed a model in which a TNFR agonist antibody binds to epitopes shared by adjacent dimers of a TNFR, thus cross-linking those dimers into a higher-order network in the presence of endogenous TNF ligand (**Figure 2**) (9). If true, this mechanism is also susceptible to a bell-shaped dose response curve, as depicted in **Figure 2**. A recent study by Yu et al. carefully investigated the relationship between affinity, FcγR binding and receptor off-rate kinetics, and demonstrated that reducing the affinity of TNFR antibody interactions was sufficient to promote increased receptor clustering and agonist function, as had been suggested previously by Ho et al. (18, 52). For both CD40 and 41BB specific antibodies, these authors demonstrated that faster off-rates improved agonist activity if the overall affinity remained approximately within the 1-300 nM range. These findings were dependent upon antibody bivalency, but only partially dependent on FcγR binding. Whether or not FcγR binding is essential is likely influenced by the specific epitope bound, and which CRD domain that epitope resides in (19, 52). Low affinity TNFR agonist mAbs could function in a model according to the one proposed by Vanamee & Faustman, but have also been shown to function in the absence of ligand *in vitro*. In either model, the low-affinity & high off-rate properties likely endowed the candidate antibody with ‘toggling’ characteristics, wherein receptor occupancy was never fully saturated because the antibodies were constantly associating and dissociating between membrane-proximal TNFR (**Figure 3**). This mechanism is unlikely to be as susceptible to a prototypical bell-shaped dose response curve in humans, as has been observed with high affinity antibodies.

Aside from the unusual bell-shaped dose response properties of TNFR agonist antibodies in humans, development of many agents has been hampered due to the emergence of dose-dependent toxicities, principally in the form of liver toxicity or cytokine release syndrome - particularly for CD40 and 41BB agonist antibodies. Liver toxicities and/or cytokine release syndrome have been reported from phase 1 clinical trials of selicrelumab, sotigalimab, mitazalimab, ChiLob7/4, and urelumab, which occurred at doses below 0.5 mg/kg, and were partially mitigated by pre-medication with corticosteroids (8, 25, 26, 47, 53–55). Another 41BB agonist antibody, utomilumab, was not found to cause liver enzyme elevations nor cytokine release syndrome, however the highest dose tested was 0.3 mg/kg and no evidence of agonist activity was reported in humans (25). The similarity in the toxicity profile of CD40 and 41BB agonist antibodies raises the question of whether these toxicities are related to CD40 or 41BB activation, a property of the agonist antibody, or a mixture of the two. Knorr et al. demonstrated that liver toxicity for a CD40 agonist antibody correlated with the strength of binding to FcγRIIB (56). Because the ‘agonist’ activity and anti-tumor activity of the CD40 antibody was also dependent upon FcγRIIB binding, toxicity and efficacy went hand-in-hand. A strategy to circumvent this issue involved direct injection of the antibody into tumors, thus avoiding adsorption in the liver through first-pass metabolism. The specific cause of toxicity, whether agonism and toxicity go hand-in-hand with one another, whether toxicity results when a threshold of TNF receptor activation is exceeded, or result from the kinetics of receptor activation cannot be determined from these clinical studies, but inferences can be made through comparison to clinical results obtained with non-antibody agonists, as discussed in the next sections.

## Clinical data from monovalent multi-specific TNFR agonist therapeutics

**Figure 4:**
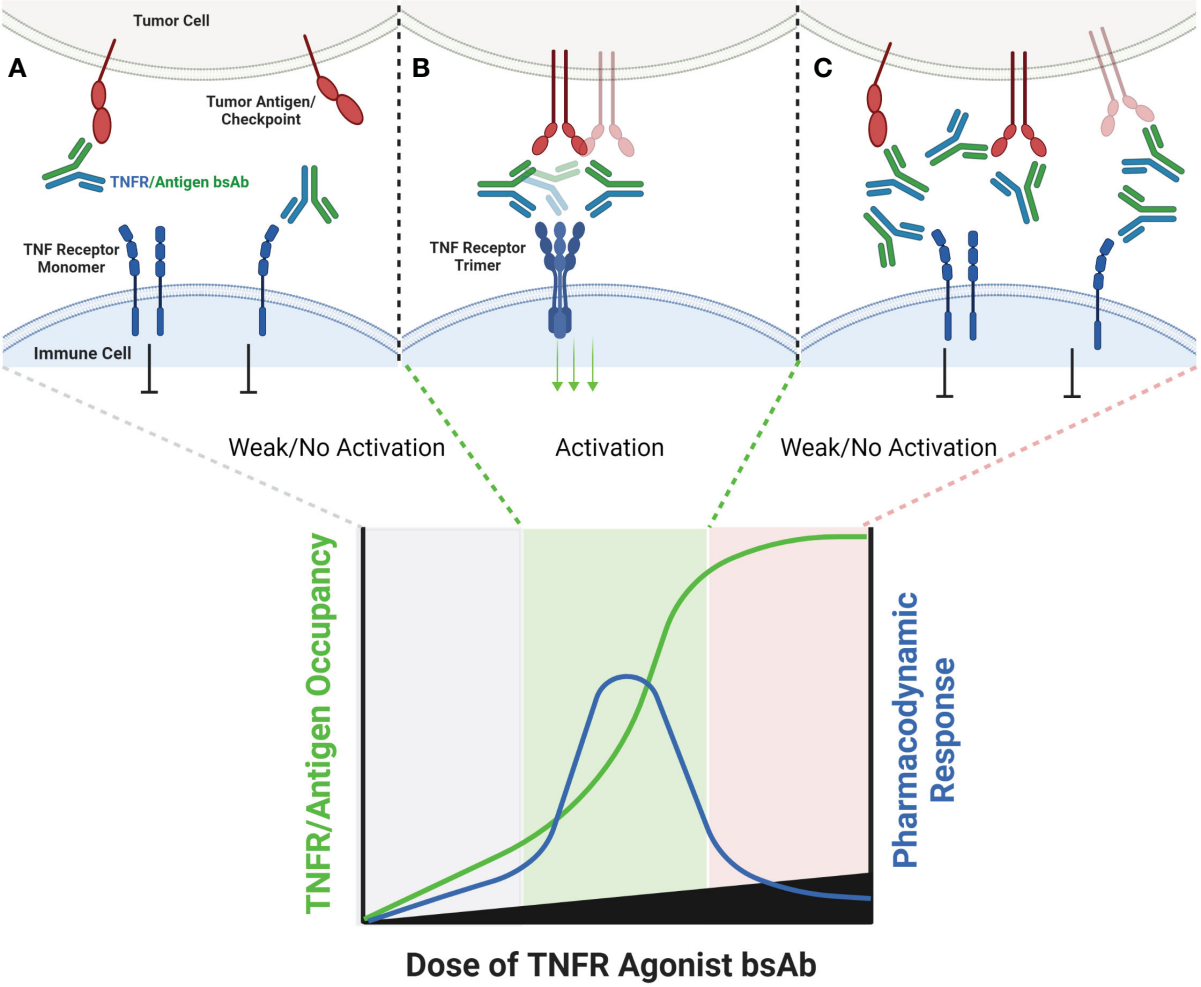
Schematic of Bispecific Antibody Mediated Clustering of TNFR. Bispecific antibodies often target an antigen expressed by a tumor cell (commonly an immune checkpoint such as PD-L1, or a tumor specific antigen such as FAP), and contain a second arm which binds a TNFR **(A)**. The TNFR binding arm of these antibodies is therefore monovalent. As the dose of the bispecific antibody is increased, the probability that multiple antibodies will cluster on the surface of an antigen positive tumor cell increases, and the probability that the monovalent TNFR binding arms from multiple antibodies will bind and approximate multiple nearby TNFR also increases **(B)**. Similar to FcγR specific mAbs, when the dose of the bispecific antibody begins to exceed approximately 50% receptor occupancy on either the tumor antigen or TNFR target, the probability that any individual antibody encounters both a free tumor antigen and TNFR target decreases, leading to a decline in TNFR clustering **(C)**.

An alternative approach to relying upon FcγR to crosslink TNFR antibody domains is to pair a combination of TNFR specific antibody domain with a tumor antigen or immune checkpoint specific antibody binding domain, thereby creating a bispecific antibody (bsAb). Examples of antibodies and antibody domain containing molecules with this structure for which clinical data are available include GEN1042 (CD40x41BB bsAb), GEN1046 (PDL1x41BB), FS222 (PDL1x41BB bsAb), PRS-343 (HER2x41BB Ab/Anticalin fusion), NM21-1480 (PDL1x41BBxHAS trispecific Ab) and MP0317 (FAPxCD40xHSA trispecific Ab). This class of agents tends to lack FcγR binding, since the underlying mechanism is proposed to rely upon antigen-specific clustering of the TNFR binding domain from multiple individual bsAbs in close proximity to one another (**Figure 4**).

In general, this class of agents lacks clinical evidence of cytokine release syndrome, and liver enzyme elevations are mild and sporadic in comparison to clinical data for bivalent antibodies directed to the same TNFR. This observation strengthens the hypothesis that the CRS and liver tox observed with TNFR agonist bivalent antibodies is largely due to FcγR driven mechanisms. GEN1042 and GEN1046 were tested across a wide dose range (0.1-400 mg and 25-1200 mg, respectively), and PK/PD models predicted a bell-shaped dose response curve, as is expected for these agents (57). Despite not requiring FcγR binding, the monovalent TNFR targeting arms of these agents still require secondary clustering via the non-TNFR targeted arm of the agent. Thus, dose levels that lead to >50% TNFR occupancy are expected to have reduced pharmacodynamic activity than those that target approximately 50% receptor occupancy, and the dose-finding complications of this mechanism discussed above are applicable. To date, limited safety, pharmacodynamic, pharmacokinetic, and clinical outcome data has been shared from clinical trials testing FS222, PRS-343, NM21-1480, and MP0317 (58–60). Each is expected to show a similar bell-shaped dose response to that of GEN1042 and GEN1046, however additional clinical data is needed to confirm this hypothesis. A longer list of agents, including YH32367 (anti-HER2/41BB), HLX35 (anti-EGFR/41BB), CB307 (anti-PSMA/41BB), RO7300490 (anti-FAP/41BB) and FS120 (anti-OX40/41BB) have been in phase 1 clinical trials for several years, however no clinical data has been publicly shared to date.

## Clinical data from trivalent, tetravalent, hexavalent and decavalent mAb-derived TNFR agonist therapeutics

Development of agonist agents which contain three or more TNFR binding domains has progressed more recently, in part due to the clinical experience obtained with the mono- and bi- valent agents described above. For category 1 TNFR, including BaffR, DR3, GITR, LTβR and TNFR1, a hexavalent agonist is likely the minimal valency to exert potent agonism (36, 61). For category 2 TNFR (41BB, BCMA, CD27, CD40, CD95, EDAR, Fn14, OX40, TACI, TNFR2, DR4 and DR5), a trimer is expected to cause signaling, however the quantum of signaling is expected to increase following assembly of hexameric or higher-order complexes (9, 32, 33, 62).

Theoretically, agonists that minimally contain a trimeric TNFR binding domain should lead to receptor activation in a soluble phase, without a requirement for cross-linking. It is tempting to speculate that these agents may not exhibit the bell-shaped dose response curves observed with monovalent and bivalent mAb formats, however emerging clinical data are suggestive of greater nuance. Of the multivalent agonists included in this discussion, some contain pre-formed ligand trimers (RO7227166 & RO7122290), some contain pre-formed ligand hexamers (SL-279252, SL-172154 & MEDI6383), and some contain a tetravalent, hexavalent or decavalent array of antibody-based TNFR binding domains. Because the pharmacodynamic activity is likely distinct between the antibody based multivalent agents, and those that contain one or more pre-formed TNF ligand trimers, the discussion between both is divided in the following sections.

**Figure 5:**
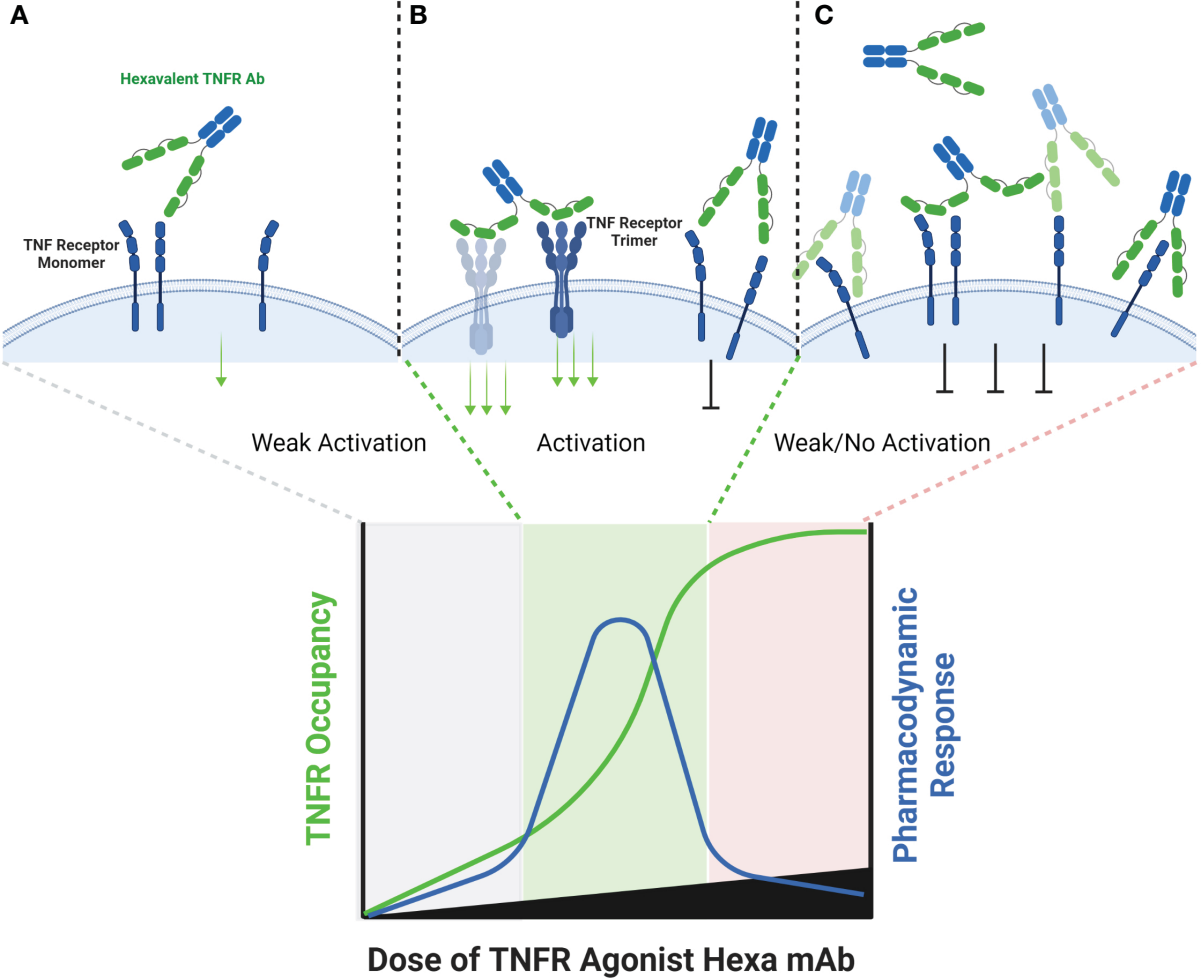
Schematic of Hexavalent mAb Binding to TNFR. Tetravalent, hexavalent and decavalent antibodies each contain sufficient TNFR binding domains to facilitate TNFR clustering in the absence of cross-linking by FcγR or a tumor antigen. Each of the binding domains of these multivalent antibodies are capable of binding to a TNFR target independently from one another, and it is possible that certain domains are more ‘exposed’ to find antigen than others, as illustrated for the distal domains **(A)**. At sub-saturating dose levels, each of the TNFR binding domains may occupy a TNFR target, and in the process cluster multiple TNFR targets into close proximity to one another, enabling activation **(B)**. Because each binding domain on an individual antibody can interact independently with a TNFR target, the theoretical maximum pharmacodynamic effect is most likely when the number of antibodies are at a 1:4, 1:6 or 1:10 ratio to the number of TNFR targets (for tetravalent, hexavalent or decavalent antibodies, respectively). When these ratios begin to be exceeded, then independent TNFR binding domains from separate antibodies are expected to compete with one another, thus reducing the probability that an individual antibody is capable of clustering the TNFR target **(C)**.

A common characteristic to agents that utilize antibody derived binding domains is that those domains are capable of binding to a TNFR regardless of whether it has pre-assembled into a trimer or hexamer in a cell membrane (**Figure 5**). The probability that a tetravalent, hexavalent or decavalent antibody leads to cross-linking of multiple TNFRs that are nearby one another in a cell membrane is undoubtedly more probable than that with a monovalent or bivalent antibody. However, if the kinetics of binding of individual antibody domains to a target are faster than the kinetics of saturation of all binding sites within individual tetravalent or hexavalent antibodies, then bell-shaped dose response curves could still be observed (**Figure 5**). The agents in clinical development that could inform on this question include; eftozanermin (ABBV-621, TRAIL-R agonist), IGM-8444 (anti-DR5), GEN1053 (anti-CD27) and INBRX-106 (anti-OX40). ABBV-621 and IGM-8444 are hexa- and decavalent DR5 agonists, respectively, mechanistically designed to trigger the death domain in DR5 to cause apoptosis in target cells (27, 63). Because the goal of therapy is to kill DR5 expressing cells, including FcγR effector function is an intended attribute of the compound. Although the pharmacodynamic data are sparse, two peripheral biomarkers of apoptosis (M30 and M65) trended lower in some of the high dose groups (≥7.5 mg/kg) relative to the lower dose groups (≤2.5 mg/kg) in a phase 1 clinical trial (63). Clinical data for IGM-8444 and GEN1053 have not yet been shared, and only qualitative comments have been made regarding the performance of INBRX-106 in a phase 1 clinical trial. INBRX-106 has an IgG1 Fc domain, and thus is capable of binding to FcγR. In a phase 1 clinical trial, toxicities were observed at a relatively low dose of 0.3 mg/kg, which led to selection of the 0.1 mg/kg dose level for further study. No pharmacokinetic, pharmacodynamic or receptor occupancy data have been shared to date.

## Clinical data from agents comprising one or more trimerized TNF ligand domains

Unlike multivalent TNFR agonists derived from a series of antibody-derived binding domains, agonists which contain pre-formed TNF ligand trimers are predicted to interact with TNF receptors in a unique manner, which potentially better reflects the native physiology of TNF ligand and receptor interactions. As described above, each individual TNFR binding domain in a multivalent TNFR targeted antibody can interact independently with a TNFR target, regardless of whether it is pre-assembled into a trimer or not. As such, there is no guarantee that each TNFR binding domain within an individual antibody molecule will become saturated before the TNFR itself becomes saturated, because there will be competition for free TNFR both between and within individual multivalent antibodies. Agents which contain pre-formed TNF ligand trimers, on the other hand, are expected to stimulate ligand-induced TNFR trimerization, and the stoichiometry of interaction is more likely to be 1:1 between individual ligand and receptor trimers. Emerging pharmacodynamic data from clinical trials supports this assertion, and is described below.

Englumafusp alfa (RO7227166) is a fusion construct comprised of a CD19-specific antibody domain fused to a trimerized extracellular domains of human 41BBL. This agent is being developed in conjunction with a CD3xCD20 T cell engager, for patients with relapsed or refractory B cell non-Hodgkin lymphoma. In a phase 1 study, RO7227166 had an acceptable safety profile across a dose range of 0.36 to 33 mg, without reaching a maximum tolerated dose (MTD). CRS was attributed to RO7227166 in just 4.8% of patients, and all of those events were Grade 1 in severity. Importantly, expansion of primed and activated T cell subsets occurred in a dose-dependent manner, without strong evidence of a bell-shaped dose response (64).

**Figure 6:**
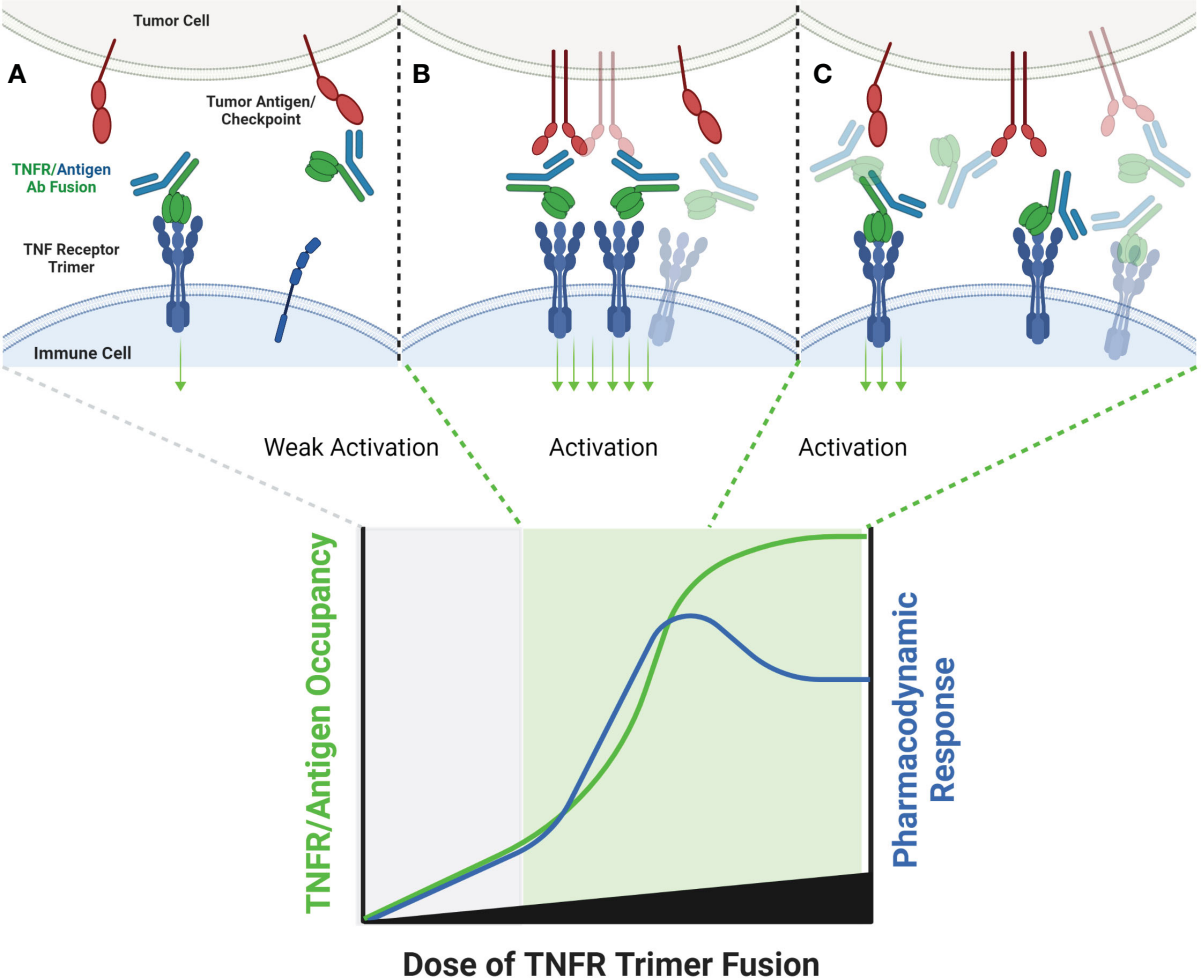
Schematic of TNF Ligand Trimer-Containing Antibody Binding to TNFR. Bispecific antibodies wherein one arm of the antibody has been replaced with a trimerized set of TNF ligand extracellular domains is capable of stimulating ligand-induced TNFR trimerization in the absence of other cross-linking or antibody clustering mechanisms, which is expected to stimulate TNFR activation even at low doses of antibody, because TNFR activation is not conditional upon clustering by the tumor antigen binding arm **(A)**. As the dose level of the antibody increases, the probability that multiple antibodies will be clustered in close proximity to one another, thus clustering multiple trimeric TNF ligand domains also increases **(B)**. If the quantum of TNFR activation is increased when the trimeric TNF ligand domains are clustered, via organizing TNFR in a higher-order network, a ‘peak’ pharmacodynamic effect may be observed at sub-saturating concentrations of antibody **(B)**. When the concentration of antibody is high enough to saturate both tumor antigen and TNFR target, the probability of TNFR network formation may decrease, leading to a tailing of the pharmacodyamic response curve, but only to a level which reflects the activity of a primarily TNFR trimer pharmacodynamic response **(C)**.

RG7827 (RO7122290) has a similar structure to RO7227166, but anchors trimerized 41BBL to FAP instead of CD19 and is being developed for patients with advanced solid tumors in combination with atezolizumab (PD-L1 mAb). A phase 1 dose escalation study evaluated RO7227166 across a dose range of 5-2000 mg, without reaching an MTD. A single case of CRS was encountered as a dose limiting toxicity, and no evidence of liver enzyme elevations were observed in the RO7122290 monotherapy arm. The pharmacodynamic changes observed in humans for RO7227166 are substantially more pronounced than those reported for urelumab or utomilumab, and include not only increases in the proportion of T cells that express Ki67 (a marker of proliferation), but also increases in the absolute numbers of T cells following treatment in a dose-dependent manner (29, 31). Further, up to ~100-fold changes were observed in the serum concentration of IFNγ post treatment, along with multi-fold increases in IL-6 and TNFα. While there were increases in each of these pharmacodynamic markers across the entire dose range in some patients, there was a trend toward higher fold-changes in the 45-260 mg group as compared to the 500-2000 mg group. In contrast to human data with the antibody agonists, pharmacodynamic responses did not return to baseline in the higher dose groups, but instead showed more variability in the magnitude of induction which came shy of the peak elevations observed in the lower dose groups. It is possible that this phenomenon is related to primarily ‘trimeric’ signaling in the high dose groups, as a result of doses high enough to independently saturate FAP and 41BBL. In the lower dose groups, the increased pharmacodynamic activity could be the result of a higher proportion of molecules of RO7227166 encountering free 41BB after binding to FAP, resulting in an ‘array’ of 41BBL trimers and a higher probability of increased signaling due to hexamer or higher-order oligomer formation (**Figure 6**).

**Figure 7:**
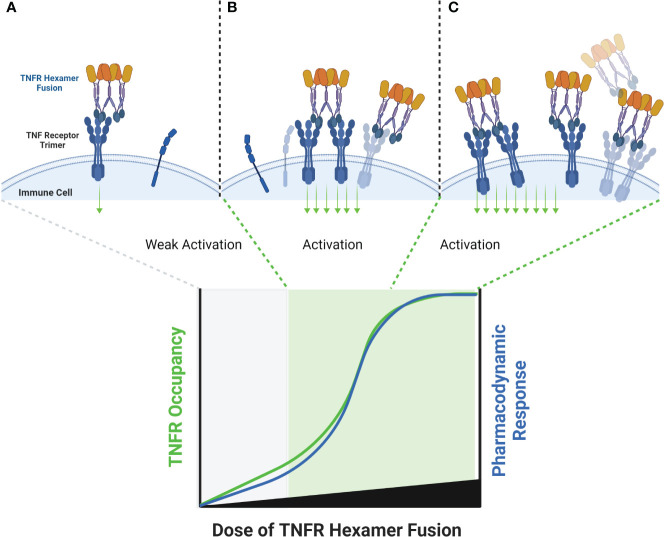
Schematic of a TNF Ligand Hexamer-Containing Fusion Protein Binding to TNFR. Fusion proteins containing two trimerized TNF ligand domains are expected to stimulate ligand-induced trimerization and TNFR hexamer network formation even at low doses of the fusion protein **(A)**. As the dose of the fusion protein increases, the probability of TNFR trimer and hexamer activation is expected to increase in proportion to the dose of the fusion protein, because the TNF ligand domains are not expected to bind TNFR monomers efficiently due to the lower avidity characteristics of the interaction **(B)**. An increasing pharmacodynamic effect is expected until the molar ratio of TNF ligand domains to trimeric TNFR is 1:1, however there is some possibility of tailing in the pharmacodynamic response if a significant proportion of the fusion proteins bind as trimers rather than hexamers, similar to the effect described in **Figure 6** **(C)**.

Two other classes of agonist therapeutics that have been tested in humans containing pre-formed TNF ligand trimers are single- and dual-side Fc fusion proteins. TNF ligands are type II membrane proteins, and fusion of the extracellular domain of a TNF ligand to an Fc domain requires a hinge-CH2-CH3-TNF ligand configuration to ensure unhindered folding and activity of the TNF ligand domain. When expressed, the quaternary structure of an Fc-TNF ligand fusion protein is influenced both by the interchain disulfide bonds in the Fc region leading to covalent dimer formation, and by non-covalent interactions in the TNF ligand domains facilitating trimer formation. The resulting structure is a hexamer consisting of a ‘dimer of trimers’ as illustrated in **Figure 7** for a dual-sided Fc fusion protein (32, 33, 36).

At least two OX40L-containing Fc fusion proteins entered clinical trials, including a single-sided Fc-OX40L (MEDI-6383) fusion and a PD1-Fc-OX40L (SL-279252) dual-sided fusion protein. Unfortunately, clinical data from a phase 1 clinical trial with MEDI-6383 has not been published. A phase 1 clinical trial testing SL-279252 in patients with a mixture of advanced solid tumors, primarily PD-1 resistant, was completed in 2023. This study examined SL-279252 across a wide dose range of 0.001 through 24 mg/kg, and was well tolerated without any treatment related grade 3 or higher adverse events and no MTD was reached. The primary pharmacodynamic finding was immediate post-dose reductions in the number of peripheral blood CD4+OX40+ T cells following each infusion, which was dose-dependent and believed to be due to migration of OX40+ T cells from the blood into tissues after activation (49). This finding was distinct from the pharmacodynamic findings in humans with OX40 agonist mAbs, where sporadic increases in the proliferation marker Ki67 were sometimes reported in subsets of CD4+ or CD8+ T cells, were not accompanied by reported changes in the actual numbers of those subsets of T cells, and generally did not provide evidence of the agonist mechanism that was predicted by pre-clinical studies (44, 45, 65, 66).

SL-172154 is a dual-sided Fc fusion protein adjoining the extracellular domains of human SIRPα and human CD40L via a mutated IgG4-derived Fc domain lacking FcγR binding. A phase 1 monotherapy dose-escalation trial was completed in patients with platinum resistant ovarian cancer, and tested SL-172154 across a dose range of 0.1 to 10 mg/kg. In contrast to prior CD40 agonist mAbs, SL-172154 had an acceptable safety profile across the dose range, with a single incidence of grade 3 LFT elevation at the 10 mg/kg dose level, and no MTD was reached. Dose-dependent infusion related reactions were common, primarily grade 1/2, but were not consistent with typical cytokine release syndrome and no elevations in IL-6 and TNFα were observed. CD40 receptor occupancy was approximately 60-80% at the 0.1 mg/kg starting dose, and full receptor occupancy and saturation was observed by the 3 mg/kg dose. The agonist activity of SL-172154 was evident post infusion with near immediate migration of CD40+ B cells and monocytes from the peripheral blood into tissues. This pharmacodynamic effect was concurrent with rapid release of cytokines and chemokines into the serum, including: IL-12, CXCL10, CCL2, CCL3, CCL4, CCL22, IL-8, IL-10 and others (30). These pharmacodynamic observations translated between species and were consistently observed in previous mouse and non-human primate studies (30, 32). This translatability has often been lacking for TNFR agonist antibodies, and in contrast to prior CD40 agonists, there was no evidence of a bell-shaped dose response for any of the pharmacodynamic findings with SL-172154. In addition, the translation of these peripheral blood findings to the tumor microenvironment was noted via a shift in myeloid cell polarization from an M2-dominated to an M1-dominated phenotype. The potency of the pharmacodynamic effects for SL-172154 exceed those reported for any prior CD40 agonist agent, and may reflect the benefit of agents containing hexamerized ligands, which potentiate ligand-induced trimerization and network formation of target TNFR (**Figure 7**).

## Conclusions and future directions

Over the past thirty years, a tremendous level of effort, investment, innovation and hope has supported the testing of many types of TNF receptor agonists in human clinical trials. Unfortunately, the resulting clinical data did not closely resemble the biology of the TNF receptor agonism predicted by pre-clinical studies, which prompted the question of whether the failure in translation was more likely a result of the intrinsic biology of TNF receptors, or of the therapeutics used to target those receptors in patients.

Some generalizations on the necessity for TNFRs to trimerize in order to signal were made throughout this review. As with most rules, these generalizations are acknowledged to have limits and special cases where they may not apply. As an example, NGF is a TNF receptor which can be activated by neurotrophin ligands, which are dimeric. In addition, the models proposed in the figures to summarize clinical data from various TNF receptor agonist agents assume that the distribution of a particular agonist agent to its potential binding partners *in vivo* are balanced. For example, **Figure 1** assumes that the TNFR agonist mAb occupies the TNFR target and Fcγ receptor targets in a roughly proportional manner. Whether or not this happens *in vivo* is influenced by multiple factors, including the binding affinity and abundance of each target. There are not any publicly available clinical data which demonstrate the relative receptor occupancy kinetics of TNFR agonist antibodies in this manner, so the models should be interpreted in a qualitative manner with these assumptions in mind.

A review of the clinical data across different TNF receptor agonist modalities reveals common themes that should be considered in advancing future agents to the clinic. These themes include the following:

1) High-affinity TNF receptor agonist antibodies which bind to Fcγ receptors have a higher likelihood of causing toxicities including cytokine release syndrome and/or liver enzyme elevation than bispecific antibodies which lack Fcγ receptor binding function.
2) Both bivalent antibodies and bispecific antibodies show bell-shaped dose response curves in humans, which likely limits the agonist potential of the modality, and creates risk that a ‘recommended phase 2 dose’ may not cause reproducible agonist effects due to variable starting frequencies of the TNF receptor expressing cells between patients, and to dynamic expression of the TNF receptor target within individual patients over time.
3) Antibody therapeutics containing three or more domains each capable of binding a TNF receptor target have a higher probability of agonist activity than mono- or bivalent antibody therapeutics, and do not require FcγR binding for function.
4) Antibody therapeutics containing three or more domains each capable of binding a TNF receptor target may still encounter bell-shaped dose response curves, similar to bispecific and bivalent antibodies, because TNF receptor saturation can occur in the absence of saturating the cross-linking potential of each antibody.
5) Agents containing pre-formed TNF ligand trimers demonstrate more potent evidence of agonist activity than antibody derived agents, potentially because those agents can facilitate ligand-dependent trimerization of TNF receptors.
6) Agents which contain multiple TNF ligand trimers demonstrate more sustained dose-dependent pharmacodynamic effects than those which require clustering by another mechanism to facilitate hexameric or higher-order TNF receptor network formation.

The consequences of the themes above in terms of safety and efficacy likely vary based on the specific TNF receptor being targeted. For 41BB and CD40 directed agents, cytokine release syndrome may prove to be more problematic than it ever will be for OX40 or GITR directed agents. Regardless, development of agents which require cross-linking for activity (either by FcγR or a target antigen) and thus exhibit bell-shaped dose response curves will be impractical because of the variable and dynamic nature of expression of TNF receptors between and within patients. Whether pre-clinical studies suggesting that this issue can be overcome by reducing the affinity of a TNF receptor agonist antibody will translate to clinical trials is unclear. The observation that tetravalent and hexavalent antibody agonists also exhibit bell-shaped dose response kinetics in humans should raise similar concerns about the ultimate agonist potential of these agents.

The pharmacodynamic effects of agents which contain at least one TNF ligand trimer demonstrate improved translation of pre-clinical to clinical findings in comparison to any of the antibody-based agonists. The ability of TNF ligand containing agonists to trigger ligand-induced trimerization of the target TNF receptor is a likely reason for this observation. The observed advantages of ligand-containing agonists are balanced by the potential drawbacks of a larger molecular format, including manufacturing efficiency, stability *in vivo*, risk of immunogenicity, and potential for decreased tissue penetration. Thus far, these issues have not limited the development of englumafusp alpha, RG7827, SL-279252 or SL-172154, however more data are needed from these and other agents to gain further confidence.

Drug developers should carefully review the structural lessons that are now available after over thirty years of clinical experimentation with different TNF receptor agonist agents. While pre-clinical studies may be very important for selecting which TNF receptor to target, the structure of the TNF receptor agonist advanced into the clinic should be made on the basis of clinical data gathered across the class of TNF receptors, rather than on the activity of a particular agonist agent in pre-clinical models.

## Author contributions

TS, GF and SD wrote and edited the manuscript. All authors contributed to the article and approved the submitted version.
